# Enantioselective cascade biocatalysis for deracemization of 2-hydroxy acids using a three-enzyme system

**DOI:** 10.1186/s12934-016-0560-1

**Published:** 2016-09-22

**Authors:** Ya-Ping Xue, Hao Zeng, Xiao-Lu Jin, Zhi-Qiang Liu, Yu-Guo Zheng

**Affiliations:** 1 Key Laboratory of Bioorganic Synthesis of Zhejiang Province, College of Biotechnology and Bioengineering, Zhejiang University of Technology, Hangzhou, 310014 People’s Republic of China; 2 Yosemade Pharmaceutical Co. Ltd., Jinhua, 321025 People’s Republic of China

**Keywords:** Coexpression, Cascade biocatalysis, Deracemization, 2-hydoxy acids

## Abstract

**Background:**

Enantiopure 2-hydroxy acids are key intermediates for the synthesis of pharmaceuticals and fine chemicals. We present an enantioselective cascade biocatalysis using recombinant microbial cells for deracemization of racemic 2-hydroxy acids that allows for efficient production of enantiopure 2-hydroxy acids.

**Results:**

The method was realized by a single recombinant *Escherichia coli* strain coexpressing three enzymes: (*S*)-2-hydroxy acid dehydrogenase, (*R*)-2-keto acid reductase and glucose dehydrogenase. One enantiomer [(*S*)-2-hydroxy acid] is firstly oxidized to the keto acid with (*S*)-2-hydroxy acid dehydrogenase, while the other enantiomer [(*R*)-2-hydroxy acid] remains unchanged. Then, the keto acid obtained reduced to the opposite enantiomer with (*R*)-2-keto acid reductase plus cofactor regeneration enzyme glucose dehydrogenase subsequently. The recombinant *E. coli* strain coexpressing the three enzymes was proven to be a promising biocatalyst for the cascade bioconversion of a structurally diverse range of racemic 2-hydroxy acids, giving the corresponding (*R*)-2-hydroxy acids in up to 98.5 % conversion and >99 % enantiomeric excess.

**Conclusions:**

In summary, a cascade biocatalysis was successfully developed to prepare valuable (*R*)-2-hydroxy acids with an efficient three-enzyme system. The developed elegant cascade biocatalysis possesses high atom efficiency and represents a promising strategy for production of highly valued (*R*)-2-hydroxy acids.

**Electronic supplementary material:**

The online version of this article (doi:10.1186/s12934-016-0560-1) contains supplementary material, which is available to authorized users.

## Background

Enantiopure 2-hydroxy acids are among the most important building blocks for synthesizing pharmaceuticals and fine chemicals [1]. For example, (*R*)-(-)-mandelic acid is widely used as an intermediate for the preparation of antibiotics, antiobesity drugs, and antitumor agents [2]. (*R*)-*o*-Chloromandelic acid is the most preferred chiral building block for the industrial synthesis of anti-thrombotic agent, a best-selling cardiovascular drug [3]. (*R*)-2-Hydroxy-4-phenylbutyric acid is an intermediate in the manufacture of angiotensin converting enzyme inhibitors [4]. (*R*)-3-Phenyllactic acid is used as a precursor for the synthesis of englitazone which has excellent hypoglycemic effect [5]. Due to their importance, many enantioselective routes for their synthesis have been developed and a great progress has been achieved in recent years. Traditionally, their industrial production mainly relies on the chemical approaches such as chemical kinetic resolution with chiral agent. However, it does not always satisfactorily work because of expensive agent, unsatisfied enantiomeric excess (*ee*) of product or low yield (e.g., <50 %).

**Scheme 1:**
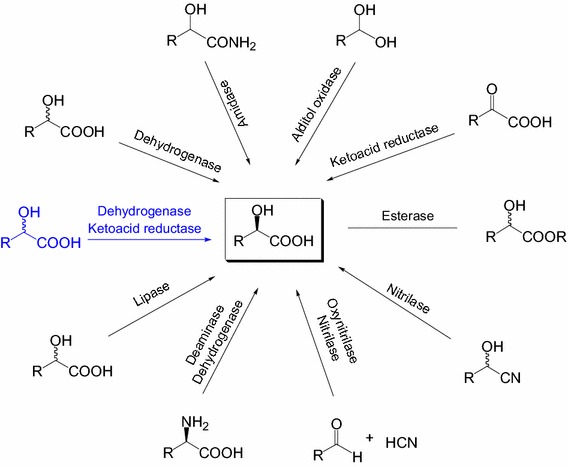
Enzymatic routes for the synthesis of enantiopure (*R*)-2-hydroxy acids

Biocatalysis is increasingly being used to develop efficient and green processes for chiral 2-hydroxy acids synthesis [6]. Several enzymatic approaches have been reported for synthesizing chiral 2-hydroxy acids in the literature [1, 7, 8], including reduction of 2-keto acids with stereoselective 2-keto acid reductase [9], enantioselective oxidation of racemic 2-hydroxy acids with 2-hydroxy acid dehydrogenase [10] or diols with alditol oxidase [11], resolution of 2-hydroxy acids with lipase [12] or esterase [1], hydrolysis of 2-hydroxynitriles with nitrilase [13, 14], enantioselective addition of HCN to aldehydes with oxynitrilase followed by nitrilase hydrolysis [15], hydrolysis of amide with amidase [16], oxidation of L-amino acids with an L-amino acid deaminase followed by asymmetric reduction of the keto acid with an 2-hydroxyisocaproate dehydrogenase [17], and deracemization of racemic 2-hydroxy acids by cascade biocatalysis [18] (Scheme 1). Among all these approaches, deracemization of racemate by cascade biocatalysis is one of the most attractive methods because it allows to completely transform a cheap racemate into a single stereoisomeric product without byproduct. Furthermore, this enantioselective cascade biocatalysis enables multistep reactions to be performed in one pot, which circumvented yield-reducing and time-consuming isolation of intermediates [19]. Recently, several types of enantioselective cascade biocatalysis have been developed for chiral synthesis [20–25], such as the deracemization of *sec*-alcohols, amines and amino acids [26–30], amination of alcohols to chiral amines [31], preparation of chiral α-hydroxy ketones from epoxides [32], and biotransformation of α-hydroxy acids into chiral α-amino acids [33, 34].

**Scheme 2:**
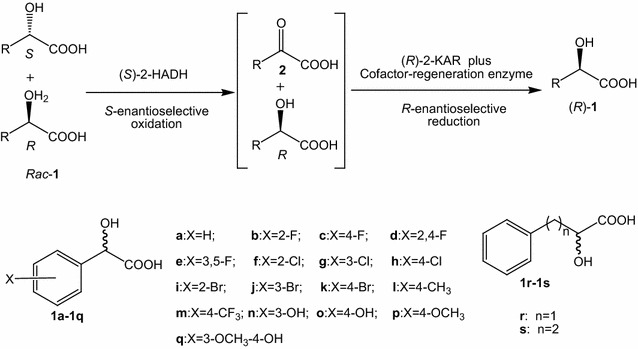
Enantioselective cascade biocatalysis for deracemization of 2-hydroxy acids to enantiopure 2-hydroxy acids via an oxidation–reduction sequence

In this work, we aim to develop an enantioselective cascade biocatalysis for deracemization of racemic 2-hydroxy acids to (*R*)-2-hydroxy acids via an oxidation–reduction sequence using a recombinant *Escherichia coli* expressing three enzymes (Scheme 2). One enantiomer [(*S*)-2-hydroxy acid] is firstly oxidized to the keto acid with enantioselective (*S*)-2-hydroxy acid dehydrogenase [(*S*)-2-HADH] while the other enantiomer [(*R*)-2-hydroxy acid] remains unchanged. The keto acid obtained is then bioreduced to the opposite enantiomer with stereoselective (*R*)-2-keto acid reductase [(*R*)-2-KAR] plus cofactor regeneration enzyme glucose dehydrogenase (GDH) subsequently. The recombinant *E. coli* strain coexpressing (*S*)-2-HADH, (*R*)-2-KAR and GDH was proven to be a promising biocatalyst. A wide range of 2-hydroxy acids can be deracemized to (*R*)-2-hydroxy acids with near-perfect stereo purity and high conversion. This method by cascade enantioselective oxidation and asymmetric reduction with a single recombinant strain represents a cheap, easy and environmental approach for synthesizing (*R*)-2-hydroxy acids from their racemates.

## Results and discussion

### Construction of recombinant *E. coli* strain expressing (S)-2-HADH

We recently established a high-throughput screening method to screen stereoselective (*S*)-2-HADH producing strains [35]. *Pseudomonas aeruginosa* CCTCC M 2011394 harboring a flavine mononucleotice (FMN)-dependent (*S*)-2-HADH, which specifically oxidizes the (*S*)-isomer of 2-hydroxy acids to 2-keto acids [36], was isolated from the soil samples. Thus, (*S*)-2-HADH may be used as the biocatalyst for the oxidation step in the designed enantioselective cascade biocatalysis. The gene of (*S*)-2-HADH from *P. aeruginosa* CCTCC M 2011394 (GenBank accession number: KU612124) was cloned and expressed in *E. coli* BL21(DE3). The recombinant *E. coli* was cultured in LB medium at 37 °C to reach an OD_600_ of 0.6 and then induced by the addition of isopropyl β-D-1-thiogalactopyranoside (IPTG) at 0.1 mM. The cells were continually grown at 28 °C, 150 rpm for 12 h. The resting cells of the recombinant *E. coli* strain (*E. coli* BL21(DE3)/pET28b-HADH) were used as biocatalysts for the enantioselective oxidation of racemic 2-hydroxy acids with *rac*-**1a** as model substrate. The result showed that the activity was lower than 5.0 U/g dry cell weight (DCW) and conversion of 2-keto acid **2a** was only 5.1 % after 2 h reaction (Table 1, entry 1). To achieve economic feasibility and competitiveness for the enantioselective oxidation of 2-hydroxy acids, it is necessary to find an promising (*S*)-2-HADH showing high activity and enantioselectivity (*E*). We adopted a genome mining strategy to search for more efficient (*S*)-2-HADHs. A pBLAST search was conducted by using (*S*)-2-HADH from *P. aeruginosa* CCTCCM 2011394 as the template in the NCBI database. Four representative (*S*)-2-HADHs from *Burkholderia xenovorans* LB400 (ABE35802.1), *P. putida* ATCC 12633 (AAC15503.1), *P. aeruginosa* NUST (AGM49308.1), *P. fluorescens* strain EBC191(AAW79575.1) were selected (Additional file 1: Figure S1). After being synthesized in vitro and cloned into pET28b, the four (*S*)-2-HADH genes were then expressed in *E. coli* BL21(DE3), respectively. *Rac*-**1a** was used as the model substrate to evaluate their activity and enantioselectivity. The results indicated that they all displayed oxidation activities. The resting cells of recombinant *E. coli* BL21(DE3) expressing the (*S*)-2-HADH from the *B. xenovorans* LB400 and *P. aeruginosa* NUST showed relatively higher activity (>90 U/g DCW) with excellent enantioselectivity (*E* > 200). After 2 h reaction, the conversions of keto acid **2a** reach 49.0 and 48.9 %, respectively, which were near theoretical conversions. However, sodium dodecyl sulfate polyacrylamide gel electrophoresis (SDS-PAGE) analysis showed that (*S*)-2-HADH from *B. xenovorans* LB400 has been expressed in partially soluble state. In the forthcoming experiments, the (*S*)-2-HADH from *P. aeruginosa* NSUT was selected for further studies. The requirement of coenzyme in the stereoselective oxidation catalyzed by (*S*)-2-HADH from *P. aeruginosa* NSUT was investigated. The activity of (*S*)-2-HADH was almost lost upon flavin removal. The activity of the apoenzyme was partly reactivated by the addition of FMN. These results confirmed that the (*S*)-2-HADH from *P. aeruginosa* NUST is a flavoprotein with FMN as cofactor. The reaction that oxidizes (*S*)-2-hydroxy acids to 2-keto acids consists of the steps involved in substrate oxidation and FMN reduction [37]. The FMN is then reoxidized by electron transfer to the oxidant. The FMN-dependent (*S*)-2-HADH family can be divided into three subgroups based on the different oxidants including oxygen, flavocytochrome b2s and ubiquinone utilized in the second oxidative half-reaction [37, 38]. Operation parameters, including optimum temperature and pH of the dehydrogenation by the recombinant *E. coli* BL21(DE3)/pET28b-HADH were investigated. The result showed that the resting cells of recombinant *E. coli* BL21(DE3)/pET28b-HADH showed high activity at 35–55 °C and pH 7.5–8.5 (Additional file 1: Figure S2). The wide ranges of optimum temperature and pH are very beneficial for the cascade biocatalysis.

**Table 1:** Catalytic performance of resting cells of recombinant *E. coli* expressing the (*S*)-2-HADH from different microorganisms

Entry	Enzyme source	Specific activity (U/g DCW)^a^	Conversion of **2a** (%)^b^	*E*
1	*P. aeruginosa* CCTCC M 2011394	<5	5.1	>200
2	*P. aeruginosa* strain NUST	107.4	48.9	>200
3	*B. xenovorans* LB400	90.2	49.0	>200
4	*P. fluorescens* strain EBC191	<5	5.2	N.D.
5	*P. putida* ATCC 12633	15.0	23.0	N.D.

*N.D.* not determined

^a^The enzyme assays were performed at 35 °C, pH 7.5 for 10 min. One unit of enzyme activity was defined as the amount of enzyme catalyzing the oxidation of substrate **1a** for producing 1.0 µmol of keto acid in 1.0 min under standard assay conditions. The substrate concentration was 20 mM

^b^The conversion of 2-keto acid was calculated when the reactions were carried out for 2 h

### Construction of recombinant *E. coli* strain coexpressing (R)-2-KAR and GDH

Stereoselective (*R*)-2-KAR could reduce prochiral 2-keto acids to produce corresponding chiral 2-hydroxy acids. The gene of (*R*)-2-KAR cloned from *Leuconostoc mesenteroides* CCTCC M 2016063 (GenBank accession number: KU612125) was expressed in *E. coli* BL21(DE3). After cultivation, the whole cells of recombinant *E. coli* BL21(DE3)/pET28b-KAR were collected and disrupted by sonication. The (*R*)-2-KAR with N-terminal his-tag in the cell free extract was purified to homogeneity by nickel affinity chromatography. The purified (*R*)-2-KAR migrated as a single band and located at the position of about 32 kDa on SDS-PAGE (Additional file 1: Figure S3), which is in agreement with the molecular mass deduced from its amino acid sequence. The purified enzyme showed little activity with NADPH but full activity with NADH, indicating an NADH-dependence. For the application of reductase, the addition of expensive cofactor often makes the bioreaction less practically feasible from the viewpoint of economic aspects. A coexpression of two enzymes in one *E. coli* cell seems to be an efficient approach to solve this problem. Thus, we introduced a GDH from *Exiguobacterium sibiricum* (WP_012369122.1) for the regeneration of the oxidized cofactor (NAD^+^). A coexpression plasmid (pCDFDuet-KAR-GDH) containing both (*R*)-2-KAR and GDH genes was constructed and transformed into *E. coli* BL21(DE3) cells. After cultivation, the whole cells of recombinant *E. coli* BL21(DE3)/pCDFDuet-KAR-GDH were collected and disrupted by sonication. The SDS-PAGE of the cell free extract of the recombinant *E. coli* showed that the coexpressed (*R*)-2-KAR and GDH were clearly visible (Additional file 1: Figure S4). The (*R*)-2-KAR and GDH located at the position of about 32 and 28 kDa on SDS-PAGE. The effects of temperature and pH on the reduction by the recombinant *E. coli* BL21(DE3)/pCDFDuet-KAR-GDH were also investigated. The resting cells of the recombinant *E. coli* exhibit high activity at 35 °C and pH 7.5 using keto acid **2a** as substrate. To test the potential of recombinant *E. coli* BL21(DE3)/pCDFDuet-KAR-GDH in chemical synthesis, various substrates were used for asymmetric reduction. With the assistance of GDH from *E. sibiricum* for cofactor regeneration, the resting cells of *E. coli* strain coexpressing (*R*)-2-KAR and GDH could reduce a wide range of prochiral 2-keto acids to corresponding (*R*)-2-hydroxy acids with >99 % *ee*. The substituents in substrates and the distance between the hydroxy group and benzene ring are the important factors to affect the catalytic ability of the biocatalyst. Among all the 2-hydroxy acids tested, substrate **2a–2m** could be efficiently reduced to (*R*)-2-**1a–1m** in >86 % conversion and >99 % *ee* within 3.5–10 h (Table 2, entries 1–13). When the OH and OCH_3_ were attached to the phenyl ring of the substrates (**2n**–**2q**) and the distance between the hydroxy group and benzene ring increased (**2r** and **2s**), the recombinant *E. coli* exhibited a relatively low activity (Table 2, entries 14–19).

**Table 2:** Reduction of keto acids to corresponding (*R*)-2-hydroxy acids by resting cells of *E. coli* BL21(DE3)/pCDFDuet-KAR-GDH

Entry	Substrate	Reaction time (h)	Conversion (%)	*ee* of (*R*)-**1** (%)
1	**2a**	3.5	95.4	>99
2	**2b**	5	97.0	>99
3	**2c**	6	92.8	>99
4	**2d**	7	89.3	>99
5	**2e**	6	90.2	>99
6	**2f**	6	99.0	>99
7	**2g**	8	90.0	>99
8	**2h**	10	87.4	>99
9	**2i**	9	96.4	>99
10	**2j**	7	91.5	>99
11	**2k**	7	86.9	>99
12	**2l**	8	87.2	>99
13	**2m**	7	88.5	>99
14	**2n**	12	26.8	>99
15	**2o**	12	22.6	>99
16	**2p**	9	76.4	>99
17	**2q**	11	25.5	>99
18	**2r**	12	21.5	>99
19	**2s**	13	14.0	>99

The reactions were performed at 35 °C in phosphate buffer (100 mM, pH 7.5) with 4 g DCW/L resting cells of *E. coli* BL21(DE3)/pCDFDuet-KAR-GDH and 20 mM substrate

### Deracemization of 2-hydroxy acids with the mixtures of recombinant *E. coli* BL21(DE3)/pET28b-HADH and *E. coli* BL21(DE3)/pCDFDuet-KAR-GDH

**Fig. 1:**
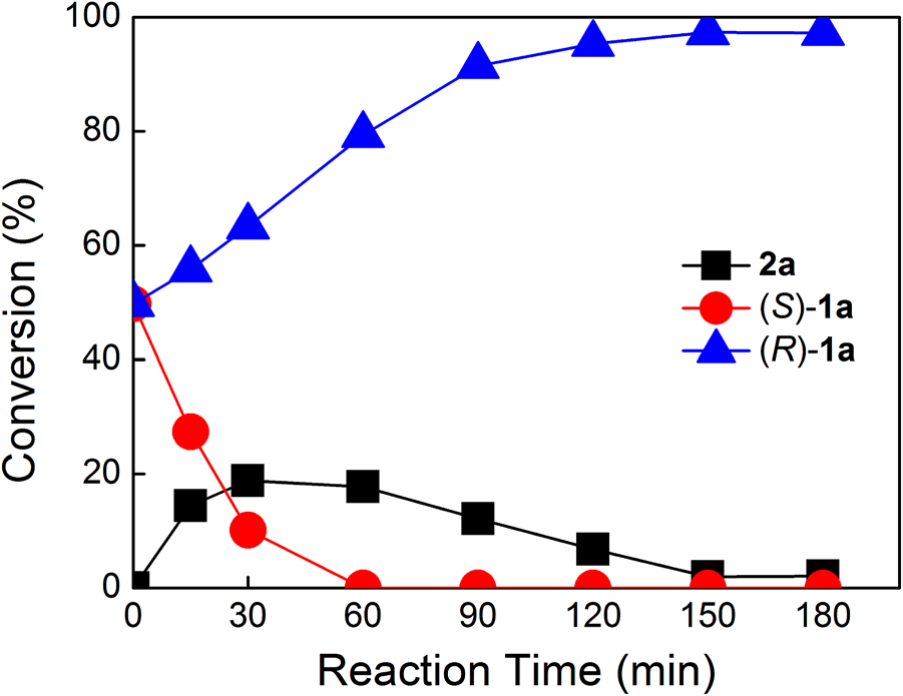
Time course of deracemization of *rac*-**1a** with the mixtures of recombinant *E. coli* strain. The freshly prepared cells of *E. coli* BL21(DE3)/pET28b-HADH and *E. coli* BL21(DE3)/pCDFDuet-KAR-GDH were mixed in 10 mL of phosphate buffer (100 mM, pH 7.5) to a cell density of 4 and 8 g DCW/L, respectively

For developing a process for deracemization of racemic 2-hydroxy acids, we coupled the asymmetric oxidation with the opposite stereoselective reduction. Recombinant *E. coli* BL21(DE3)/pET28b-HADH and *E. coli* BL21(DE3)/pCDFDuet-KAR-GDH were cultivated, separately, to achieve the resting cells. The cells of the mixed two strains were designed as the catalytic system. The enantioselective cascade biocatalysis for deracemization of **1a**–**1s** was carried out by a one-pot strategy. The conversion, *ee* of products and reaction time were detected (Table 3). The result showed that the (*R*)-isomers of substrates (**1a–1m**) were obtained in high conversions (>90 %) with >99 % *ee*. In the case of **1n–1s** (Table 3, entries 14–19), (*R*)-2-hydroxy acids were obtained with relative low conversion (34.6–76.4 %) within 6 h. For the **1p–1q** (Table 3, entry 16–17), (*R*)-2-hydroxy acids were obtained with high enantiomeric excess (>99 %), which indicated that the (*S*)-isomer of 2-hydroxy acids were completely oxidized. The lower conversion might be due to the accumulation of 2-keto acids. Figure 1 shows a typical deracemization progress for the preparation of (*R*)-**1a** from **1a** with the mixtures of recombinant *E. coli* BL21(DE3)/pET28b-HADH and *E. coli* BL21(DE3)/pCDFDuet-KAR-GDH. Oxidation was the faster step in the overall reaction because **2a** was accumulated in the cascade reaction. After 150 min reaction, the conversion to (*R*)-**1a** from racemate was 97 %.

**Table 3:** Deracemization of 2-hydroxy acids with the mixtures of *E. coli* BL21(DE3)/pET28b-HADH and *E. coli* BL21(DE3)/pCDFDuet-KAR-GDH

Entry	Substrate	Reaction time (h)	Conversion (%)	*ee* of (*R*)-**1** (%)
1	*rac*-**1a**	4	94.9	>99
2	*rac*-**1b**	6	94.5	>99
3	*rac*-**1c**	6	93.2	>99
4	*rac*-**1d**	4	95.1	>99
5	*rac*-**1e**	4	96.4	>99
6	*rac*-**1f**	4	97.3	>99
7	*rac*-**1g**	4	97.6	>99
8	*rac*-**1h**	2	95.5	>99
9	*rac*-**1i**	4	96.8	>99
10	*rac*-**1j**	2	94.5	>99
11	*rac*-**1k**	4	96.3	>99
12	*rac*-**1l**	2	96.4	>99
13	*rac*-**1m**	4	94.8	>99
14	*rac*-**1n**	6	35.5	81.9
15	*rac*-**1o**	6	34.6	71.8
16	*rac*-**1p**	6	59.5	>99
17	*rac*-**1q**	6	42.7	>99
18	*rac*-**1r**	6	76.4	63.3
19	*rac*-**1s**	6	58.3	60.1

Reactions were performed in one pot at 35 °C in phosphate buffer (100 mM, pH 7.5) with 4 g DCW/L resting cells of *E. coli* BL21 (DE3)/pET28b-HADH, 8 g DCW/L resting cells of *E. coli* BL21 (DE3)/pCDFDuet-KAR-GDH and 20 mM substrate

### Construction of recombinant *E. coli* coexpressing (S)-2-HADH, (R)-2-KAR and GDH for deracemization of 2-hydroxy acids by cascade biocatalysis

**Fig. 2:**
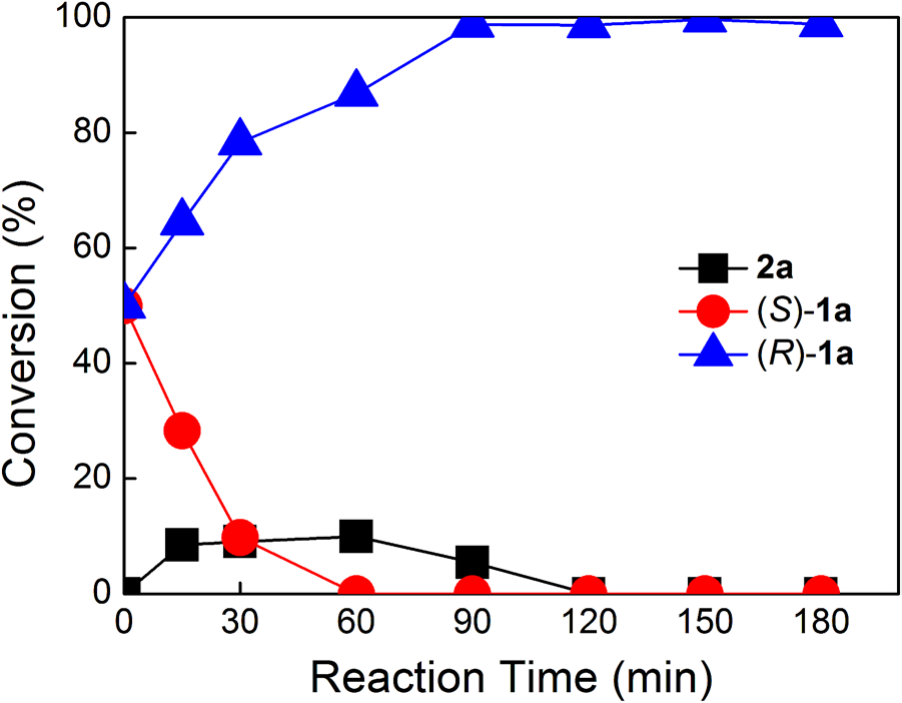
Time course of deracemization of *rac*-**1a** with a single recombinant *E. coli* strain. The freshly prepared cells of *E. coli* BL21(DE3)/pET28b-HADH/pCDFDuet-KAR-GDH were resuspended in 10 mL phosphate buffer (100 mM, pH 7.5) to a cell concentration of 8 g DCW/L

In order to avoid the respective cultivation of the two recombinant strains and reduce the cell concentration in the cascade biocatalysis, we attempted to use a single recombinant strain coexpressing (*S*)-2-HADH, (*R*)-2-KAR and GDH for the cascade biocatalysis. The recombinant *E. coli* strain expressing all the necessary enzymes was constructed. The pET28b-HADH and pCDFDuet-KAR-GDH with different antibiotic selection were introduced to *E. coli* BL21(DE3). The recombinant strain was selected with LB agar plates containing streptomycin and kanamycin. A three-enzyme coexpression strain *E. coli* BL21(DE3)/pET28b-HADH/pCDFDuet-KAR-GDH was screened. The SDS-PAGE of the cell free extract of the recombinant *E. coli* showed that the coexpressed (*S*)-2-HADH, (*R*)-2-KAR and GDH were clearly visible (Additional file 1: Figure S5). The constructed three-enzyme system was used for one-pot cascade biocatalysis. The recombinant *E. coli* BL21(DE3)/pET28b-HADH/pCDFDuet-KAR-GDH was cultured to achieve the resting cells. The cascade oxidation–reduction reaction catalyzed by the resting cells of the recombinant *E. coli* were performed at 35 °C and pH 7.5 (Table 4). The results showed that most of those substrates (**1a–1m**) can be obtained in up to 98.5 % conversion and >99 % *ee* in a shorter reaction time as compared to the mixtures of two recombinant *E. coli*. The reason for this may be that the multienzyme in one recombinant strain avoid the transfer of substrates in different cells. Figure 2 shows a typical time course for production of (*R*)-**1a** from racemic **1a** with a single recombinant *E. coli* BL21(DE3)/pET28b-HADH/pCDFDuet-KAR-GDH expressing all the necessary enzymes. After 100 min reaction, (*S*)-**1a** was almost completely converted to (*R*)-**1a**. The results confirmed that the three-enzyme coexpressing system was more efficient.

**Table 4:** Deracemization of 2-hydroxy acids with recombinant *E. coli* coexpressing (S)-2-HADH, (*R*)-2-KAR and GDH

Entry	Substrate	Reaction time (h)	Conversion (%)	*ee* of (*R*)-**1** (%)
1	*rac*-**1a**	2	95.2	>99
2	*rac*-**1b**	2	92.7	>99
3	*rac*-**1c**	2	95.8	>99
4	*rac*-**1d**	4	95.9	>99
5	*rac*-**1e**	2	98.5	>99
6	*rac*-**1f**	2	98.2	>99
7	*rac*-**1g**	2	97.4	>99
8	*rac*-**1h**	2	97.7	>99
9	*rac*-**1i**	2	96.6	>99
10	*rac*-**1j**	2	97.1	>99
11	*rac*-**1k**	2	97.4	>99
12	*rac*-**1l**	2	96.9	>99
13	*rac*-**1m**	2	97.7	>99
14	*rac*-**1n**	6	37.4	91.5
15	*rac*-**1o**	6	33.9	73.6
16	*rac*-**1p**	6	76.9	>99
17	*rac*-**1q**	6	40.5	>99
18	*rac*-**1r**	6	90.7	85.8
19	*rac*-**1s**	6	82.4	78.6

Reactions were performed in one pot at 35 °C in phosphate buffer (100 mM, pH 7.5) with 8 g DCW/L resting cells of *E. coli* BL21(DE3)/pET28b-HADH/pCDFDuet-KAR-GDH and 20 mM substrate

## Conclusions

In summary, a cascade biocatalysis was successfully developed to prepare valuable (*R*)-2-hydroxy acids with an efficient three-enzyme system, which was constructed by coexpressing (*S*)-2-HADH, (*R*)-2-KAR and GDH. The recombinant *E. coli* strain coexpressing the three enzymes was proven to be a promising biocatalyst for the cascade bioconversion of a structurally diverse range of racemic 2-hydroxy acids, giving the corresponding (*R*)-2-hydroxy acids in up to 98.5 % conversion and >99 % *ee*. The developed elegant cascade biocatalysis possesses high atom efficiency and represents a promising strategy for production of highly valued (*R*)-2-hydroxy acids.

## Methods

### Materials

**1a**–**1s**, (*R*)-**1a**, (*S*)-**1a** and benzoyl formic acid were provided from J&K Chemical Co., Ltd. (Shanghai, China). All other chemicals used were of analytical grade and commercially available. DNA purification kits, restriction endonucleases, T4 DNA ligase, *Pfu* DNA polymerase, and *Taq* DNA polymerase were purchased from Shenergy Biocolor BioScience and Technology Company (Shanghai, China). The pGEM-T (Promega, Madison, WI, USA) was used as cloning vector. The pETDuet-1 and pET28b (Novagen, Darmstadt, Germany) were used as expression vector.

### Construction of recombinant *E. coli* BL21(DE3)/pET28b-HADH

The gene encoding (*S*)-2-HADH from *P. aeruginosa* CCTCC M 2011394 was amplified via PCR with a series of primers (F-1 GGTGAAACACAACCGCGA; R-1 AGGGCATCCAATCTGGGC, F-2 CTATGGTTCCAGCTCTATGTG; R-2 AGTTGGCGACCGCCGTG, F-3 CATGCCGCAACTGGCCAA; R-3 TGCGGTCGATATCCGCTTT). (*S*)-2-HADH gene was amplified via PCR with primers (F- GGAATTCCATATGATGATCATTTCCGCTTCCACC, R- CCCAAGCTTTCAGGCGCCCAGTTCGCGGACCA) designed according to the sequence similarity. The PCR product was digested with *Nde*I and *Hin*dIII and ligated into pET28b. The recombinant plasmids were transformed into *E. coli* BL21(DE3) for expression. A pBLAST search was conducted by using (*S*)-2-HADH from *P. aeruginosa* CCTCCM 2011394 as the template in the NCBI database. The (*S*)-2-HADH from *B. xenovorans* LB400 (ABE35802.1), *P. putida* (AAC15503.1), *P. aeruginosa* NUST (AGM49308.1), *P. fluorescens* strain EBC191 (AAW79575.1) were selected. Nucleotide sequences of (*S*)-2-HADHs from these strains were synthesized using the polymerase chain reaction assembly method [39]. The coding genes were ligated into pET28b and expressed in *E. coli* BL21(DE3). For the selection of *E. coli* BL21(DE3) transformants, 50 μg/mL kanamycin was added to the Luria–Bertani (LB) medium (5 g yeast extract, 10 g tryptone, and 10 g NaCl in 1 L of distilled water). The requirement of coenzyme in the stereoselective oxidation catalyzed by (*S*)-2-HADH from *P. aeruginosa* NSUT was investigated according to the method as described previously [40].

### Construction of recombinant *E. coli* BL21(DE3)/pCDFDuet-KAR-GDH

The gene of (*R*)-2-KAR from *L. mesenteroides* CCTCC M 2016063 was amplified via PCR with primers (F-AGGCCATGGGTAAAATCGCAATTGCCG, R-AATCTCGAGGATCTCGAAGTTCTCTTGC). The gene of GDH from *E. sibiricum* (WP_012369122.1) was synthesized in vitro. The genes of (*R*)-2-KAR and GDH were cloned and inserted into the coexpression vector pCDFDuet. The pCDFDuet-KAR-GDH was transformed into *E. coli* BL21(DE3) for expression. For the selection of *E. coli* BL21(DE3) transformants (*E. coli* BL21(DE3)/pCDFDuet-KAR-GDH), 50 μg/mL streptomycin was added to the LB medium.

### Construction of recombinant *E. coli* BL21(DE3)/pET28b-HADH/pCDFDuet-KAR-GDH

pET28b-HADH and pCDFDuet-KAR-GDH with different antibiotic selection were transformed into *E. coli* BL21(DE3). The recombinant strain (*E. coli* BL21(DE3)/pET28b-HADH/pCDFDuet-KAR-GDH) was selected with LB agar plates containing 50 μg/mL streptomycin and 50 μg/mL kanamycin.

### Microorganisms and culture conditions

*P. aeruginosa* CCTCCM 2011394 was cultured at 30 °C in rich medium containing 10 g glucose, 10 g yeast extract, 2.5 g K_2_HPO_4_·3H_2_O, 2.5 g KH_2_PO_4_, 0.2 g MgSO_4_·7 H_2_O, 0.03 g FeSO_4_·7 H_2_O and 1.0 g NaCl in 1 L of distilled water (pH 7.0). *L. mesenteroides* CCTCC M 2016063 was cultured at 30 °C in complete medium containing 10 g glucose, 10 g yeast extract, 10 g tryptone, 5 g NaCl and 5 g beef extract in 1 L of distilled water (pH 7.0). *E. coli* BL21(DE3) (Novagen, Darmstadt, Germany) were used for gene expression. The *E. coli* strains were grown at 37 °C in LB medium. The recombinant *E. coli* strains were grown at 37 °C in LB medium containing appropriate antibiotics (50 μg/mL streptomycin, or 50 μg/mL kanamycin, or both) to reach an OD_600_ of 0.6 and then induced by adding IPTG at 0.1 mM. The strains were cultured continually at 28 °C, 150 rpm for 12 h. The whole cells were collected by centrifugation at 9000×*g* under 4 °C for 10 min, washed twice with 100 mM phosphate buffer (pH 7.5) for activity test and biotransformation.

### Cascade deracemization of 2-hydroxy acids with mixture of the resting cells of recombinant *E. coli* BL21(DE3)/pET28b-HADH and *E. coli* BL21(DE3)/pCDFDuet-KAR-GDH

The freshly prepared whole cells of *E. coli* BL21(DE3)/pET28b-HADH and *E. coli* BL21(DE3)/pCDFDuet-KAR-GDH were suspended in 10 mL of phosphate buffer (100 mM, pH 7.5) to a cell concentration of 4 and 8 g DCW/L, respectively. Racemic 2-hydroxy acids **1a–1s** was added to the mixture at a final concentration of 20 mM. The mixtures were shaken at 35 °C and 150 rpm in 50-mL flasks. Samples were taken at regular intervals and the reactions were terminated through centrifugation (12,000×*g*, 4 °C, 5 min). The conversion and *ee* of products were determined by chiral high-performance liquid chromatographic (HPLC) method.

### Cascade deracemization of 2-hydroxy acids with the resting cells of recombinant *E. coli* BL21(DE3)/pET28b-HADH/pCDFDuet-KAR-GDH

The freshly prepared cells of *E. coli* BL21(DE3)/pET28b-HADH/pCDFDuet-KAR-GDH were resuspended to a cell concentration of 8 g DCW/L in 10 mL phosphate buffer (100 mM, pH 7.5) containing 20 mM **1a–1s**. The mixtures were shaken at 35 °C and 150 rpm in 50-mL flasks. Samples were taken at regular intervals and the reactions were terminated through centrifugation (12,000×*g*, 4 °C, 5 min). The conversion and *ee* values were determined by chiral HPLC method.

### Analytical methods

The determination of (*R*)-**1a–1s**, (*S*)-**1a–1s** and **2a–2s** was performed by chiral HPLC equipped with a chiral column (Chirobiotic^TM^ R 250 × 4.6 mm, particle size 5 μm, Sigma, USA). The flow rate was set at 1.0 mL/min. The mobile phase was composed of 0.5 % NH_4_OH-CH_3_OH (10:90, v/v). The eluate was monitored at 215 nm. Enantioselectivity (*E*) was calculated from conversion and *ee* as described previously [41].

## Additional file

10.1186/s12934-016-0560-1 Amino acid sequences multiple alignment of 2-HADH from *P. aeruginosa* CCTCC M 2011394, *B. xenovorans* LB400, *P. putida* ATCC 12633, *P. aeruginosa* NUST, *P. fluorescens* strain EBC191. **Figure S2.** Optimization of biooxidation reaction conditions by resting cells of recombinant *E. coli* BL21(DE3)/pET28b-HADH. (A) Effect of temperature on the biooxidation. The optimum temperature was determined over the range from 25 and 65 °C. The recombinant *E. coli* BL21(DE3)/pET28b-HADH showed high activity at 35–55 °C, and at higher temperatures the activity began to decrease significantly. (B) Effect of pH on the biooxidation; The optimum pH on the oxidation reaction was determined over the range from 6.0 and 9.0. When the pH was below 7.5 or over 8.5, the enzyme activity decreased dramatically. **Figure S3.** SDS-PAGE analysis of the expressed (*R*)-2-KAR in recombinant *E. coli* BL21(DE3)/pET28b-KAR. 1. Markers; 2. Cell-free extract of recombinant *E. coli* BL21(DE3)/pET28b-KAR; 3. Purified (*R*)-2-KAR from *E. coli* BL21(DE3)/pET28b-KAR (~32 kDa). **Figure S4.** SDS-PAGE analysis of the expressed GDH in recombinant *E. coli* BL21(DE3)/pET28b-GDH and coexpressed (*R*)-2-KAR and GDH in recombinant *E. coli* BL21(DE3)/pCDFDuet-KAR-GDH. 1. *E. coli* BL21(DE3)/pCDFDuet-KAR-GDH with 0.1 mM IPTG. The upper arrow indicated (*R*)-2-KAR (~32 kDa) and the lower arrow represented GDH (~28 kDa).2. *E. coli* BL21(DE3)/pCDFDuet-KAR-GDH with 0 mM IPTG; 3. *E. coli* BL21(DE3)/pET28b-GDH with 0.1 mM IPTG; 4. *E. coli* BL21(DE3)/pET28b-GDH with 0 mM IPTG; 5.Empty plasmid of pET28b with 0 mM IPTG. 6. Markers. **Figure S5.** SDS-PAGE analysis of the coexpressed (S)-2-HADH, (*R*)-2-KAR and GDH in recombinant *E. coli* BL21(DE3)/pET28b-HADH/pCDFDuet-KAR-GDH. 1. Markers; 2. Cell-free extract of recombinant *E. coli* BL21(DE3)/pET28b-HADH/pCDFDuet-KAR-GDH. The upper arrow indicated (*S*)-2-HADH (~42 kDa), the middle arrow indicated (*R*)-2-KAR (~32 kDa) and the lower arrow represented GDH (~28 kDa).

## Abbreviations

(*S*)-2-HADH: (*S*)-2-hydroxy acid dehydrogenase
(*R*)-2-KAR: (*R*)-2-keto acid reductase
GDH: glucose dehydrogenase
FMN: flavine mononucleotice
DCW: dry cell weight
SDS-PAGE: sodium dodecyl sulfate polyacrylamide gel electrophoresis
IPTG: isopropyl β-d-1-thiogalactopyranoside
