# Supplementary material for: Enantioselective cascade biocatalysis for deracemization of 2-hydroxy acids using a three-enzyme system
Source: Microb Cell Fact. 2016 Sep 22;15:162. doi: 10.1186/s12934-016-0560-1 (PMC5034429; doi:10.1186/s12934-016-0560-1)
Supplement: Supplementary file 1 — 10.1186/s12934-016-0560-1 Amino acid sequences multiple alignment of 2-HADH from P. aeruginosa CCTCC M 2011394, B. xenovorans LB400, P. putida ATCC 12633, P. aeruginosa NUST, P. fluorescens strain EBC191. Figure S2. Optimization of biooxidation reaction conditions by resting cells of recombinant E. coli BL21(DE3)/pET28b-HADH. (A) Effect of temperature on the biooxidation. The optimum temperature was determined over the range from 25 and 65 °C. The recombinant E. coli BL21(DE3)/pET28b-HADH showed high activity at 35–55 °C, and at higher temperatures the activity began to decrease significantly. (B) Effect of pH on the biooxidation; The optimum pH on the oxidation reaction was determined over the range from 6.0 and 9.0. When the pH was below 7.5 or over 8.5, the enzyme activity decreased dramatically. Figure S3. SDS-PAGE analysis of the expressed (R)-2-KAR in recombinant E. coli BL21(DE3)/pET28b-KAR. 1. Markers; 2. Cell-free extract of recombinant E. coli BL21(DE3)/pET28b-KAR; 3. Purified (R)-2-KAR from E. coli BL21(DE3)/pET28b-KAR (~32 kDa). Figure S4. SDS-PAGE analysis of the expressed GDH in recombinant E. coli BL21(DE3)/pET28b-GDH and coexpressed (R)-2-KAR and GDH in recombinant E. coli BL21(DE3)/pCDFDuet-KAR-GDH. 1. E. coli BL21(DE3)/pCDFDuet-KAR-GDH with 0.1 mM IPTG. The upper arrow indicated (R)-2-KAR (~32 kDa) and the lower arrow represented GDH (~28 kDa).2. E. coli BL21(DE3)/pCDFDuet-KAR-GDH with 0 mM IPTG; 3. E. coli BL21(DE3)/pET28b-GDH with 0.1 mM IPTG; 4. E. coli BL21(DE3)/pET28b-GDH with 0 mM IPTG; 5.Empty plasmid of pET28b with 0 mM IPTG. 6. Markers. Figure S5. SDS-PAGE analysis of the coexpressed (S)-2-HADH, (R)-2-KAR and GDH in recombinant E. coli BL21(DE3)/pET28b-HADH/pCDFDuet-KAR-GDH. 1. Markers; 2. Cell-free extract of recombinant E. coli BL21(DE3)/pET28b-HADH/pCDFDuet-KAR-GDH. The upper arrow indicated (S)-2-HADH (~42 kDa), the middle arrow indicated (R)-2-KAR (~32 kDa) and the lower arrow represented GDH (~28 kDa). [file 12934_2016_560_MOESM1_ESM.docx]

**Additional file 1**

Additional file 1: Figure S1 Amino acid sequences multiple alignment of 2-HADH from *P.aeruginosa* CCTCC M 2011394, *B. xenovorans* LB400, *P.putida* ATCC 12633, *P.aeruginosa* NUST, *P.fluorescens* strain EBC191.

**Additional file 1: Figure S2** Optimization of biooxidation reaction conditions by resting cells of recombinant *E. coli* BL21(DE3)/pET28b-HADH. (A) Effect of temperature on the biooxidation. The optimum temperature was determined over the range from 25 and 65°C. The recombinant *E. coli* BL21(DE3)/pET28b-HADH showed high activity at 35°C-55°C, and at higher temperatures the activity began to decrease significantly. (B) Effect of pH on the biooxidation; The optimum pH on the oxidation reaction was determined over the range from 6.0 and 9.0. When the pH was below 7.5 or over 8.5, the enzyme activity decreased dramatically.


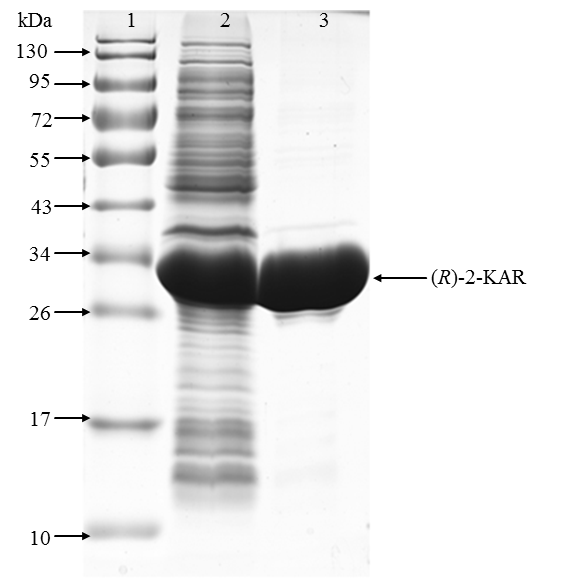


**Additional file 1: Figure S3** SDS-PAGE analysis of the expressed (*R*)-2-KAR in recombinant *E. coli* BL21(DE3)/pET28b-KAR. 1. Markers; 2. Cell-free extract of recombinant *E. coli* BL21(DE3)/pET28b-KAR; 3. Purified (*R*)-2-KAR from *E. coli* BL21(DE3)/pET28b-KAR (~32kDa).


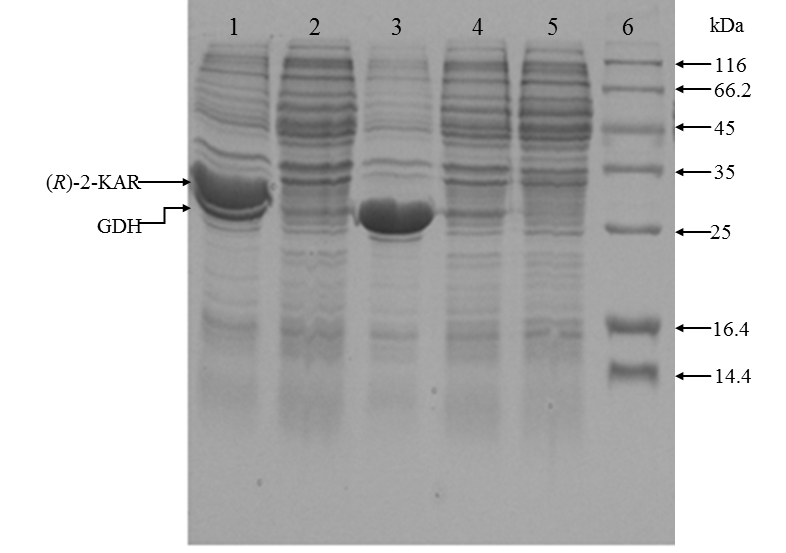


Additional file 1: Figure S4. SDS-PAGE analysis of the expressed GDH in recombinant *E. coli* BL21(DE3)/pET28b-GDH and coexpressed (*R*)-2-KAR and GDH in recombinant *E. coli* BL21(DE3)/pCDFDuet-KAR-GDH. 1. *E. coli* BL21(DE3)/pCDFDuet-KAR-GDH with 0.1 mM IPTG. The upper arrow indicated (*R*)-2-KAR (~32kDa) and the lower arrow represented GDH (~28kDa).2. *E. coli* BL21(DE3)/pCDFDuet-KAR-GDH with 0 mM IPTG; 3. *E. coli* BL21(DE3)/pET28b-GDH with 0.1 mM IPTG; 4. *E. coli* BL21(DE3)/pET28b-GDH with 0 mM IPTG; 5.Empty plasmid of pET28b with 0 mM IPTG. 6. Markers.


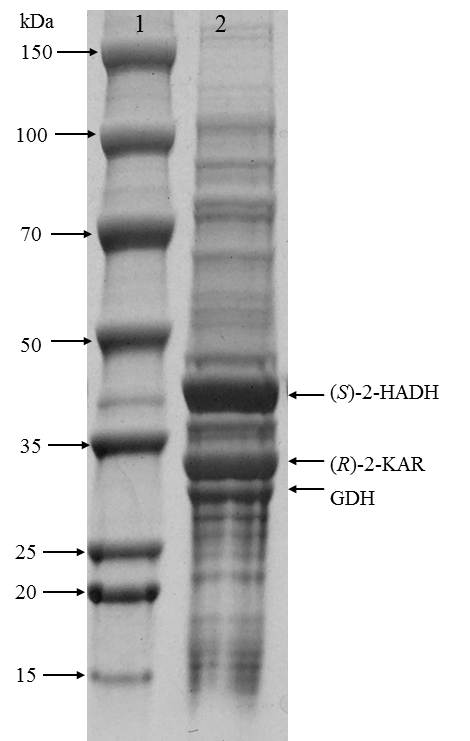


**Additional file 1: Figure S5.** SDS-PAGE analysis of the coexpressed (S)-2-HADH, (*R*)-2-KAR and GDH in recombinant *E. coli* BL21(DE3)/pET28b-HADH/pCDFDuet-KAR-GDH. 1. Markers; 2. Cell-free extract of recombinant *E. coli* BL21(DE3)/pET28b-HADH/pCDFDuet-KAR-GDH. The upper arrow indicated (*S*)-2-HADH (~42 kDa), the middle arrow indicated (*R*)-2-KAR (~32 kDa) and the lower arrow represented GDH (~28 kDa).
